# Inter-individual Variability of Coumarin 7-hydroxylation (CYP2A6 activity) in an Iranian Population

**Published:** 2013-04

**Authors:** Mohammad Hassanzadeh Khayyat, Nasser Vahdati Mashhadian, Saeed Eghbal, Navid Jalali

**Affiliations:** 1 Drug research center, Faculty of Pharmacy, Mashhad University of Medical Sciences, Mashhad, 91775-1365, Iran; 2 Medical Toxicology Research Centre, Faculty of Medicine and Department of Pharmacodynamy and Toxicology, Faculty of Pharmacy, Mashhad University of Medical Sciences, Mashhad, 91775-1365, Iran

**Keywords:** CYP2A6, Coumarin, Fluorometry, Iran, Polymorphism, 7-hydroxycoumarin

## Abstract

***Objective(s):*** Coumarin hydroxylase (CYP2A6) is a polymorphic enzyme, and during the last decade has received a lot of attention because it is the principle human nicotine *C*-oxidase, which activates a number of procarcinogens, and metabolizes drugs.

***Materials and Methods:*** 150 healthy Iranian volunteers, (96 male, 54 female) aged 19 to 63 years old, were given 5 mg coumarin orally after an overnight fast. Urine samples were collected before drug administration and 2, 4 and 8 h after that. The extent and rate of the formation of 7-OH-coumarin (7OHC) was determined by the urinary excretion of the metabolite as measured by the fluorometric method.

***Results:*** The proportion of 7OHC excreted during the first 2 hr compared to the 7OHC excretion at 8 h was the constant and stable individual characteristic for the rate of the formation of 7OHC (2 hr coumarin test). The total amount of 7OHC formed was 32.7±12.4% (mean±SD) of the given dose. On average, 66.0% of 7OHC formed was excreted in 2 hr. No clear-cut polymorphism in the rate of 7OHC formation was found. The total amounts of 7OHC excreted were significantly lower in females. Also, the rate and amount of coumarin metabolism in Iranian population were lower than Turkish and Finnish populations.

***Conclusion:*** Because of the importance of enzyme activity in drug metabolism, caution should be exercised in prescribing compounds metabolized by CYP2A6 enzyme in Iranian population.

## Introduction

There is a pronounced inter-individual and interethnic variability in the levels and activities of many drug-metabolizing enzymes. This could cause inter-individual differences in sensitivities to the effects and toxicity of many clinically used drugs and environmental compounds. In many cases, the basis for this variability is genetic polymorphism in the genes encoding these enzymes (1). Cytochrome P450s (CYPs) are a family of heme-containing enzymes implicated in the metabolism of xenobiotics in the body. Five CYPs (CYP1A2, 2C9, 2C19, 2D6, and 3A4) are responsible for approximately 90% of CYP-mediated drug metabolism in the body, with the remaining CYP enzymes being involved in steroidogenesis and fatty acid oxidation. CYPs catalyze the hydroxylation, epoxidation, and N-, S-, and O-demethylation of drug molecules, allowing for second-phase metabolism and excretion from the body (2, 3). CYP2A6, first identified as the human coumarin 7-hydroxylase, is a polymorphic member of CYP family of enzymes (4, 5). It constitutes 5-10% of the total microsomal CYPs of human liver (6). This enzyme is predominantly expressed in the liver (7) and is responsible for the clearance of many drugs and environmental chemicals. It is also the most important P450 responsible for nicotine C-oxidation and metabolizes cigarette nitrosamines as well (8).

Coumarin is a naturally occurring plant compound with a high safety profile. No adverse effects of coumarin have been reported in susceptible species in response to doses which are more than 100 times the maximum human daily intake (9). Because of its certain biochemical properties, coumarin has been proposed to be used in clinical medicine, as a macrophage activator in the treatment of certain carcinomas (10, 11). It is mainly metabolized by CYP2A6 and is widely used as a probe for the determination of activity of the enzyme (12, 13) and in polymorphism studies (4, 14, 15).

Coumarin 7-hydroxylation is catalyzed by a high-affinity CYP2A6 enzyme in human liver microsomes. CYP2A6 is the only enzyme catalyzing this reaction and consequently the formation of 7-hydroxycoumarin can be used as ‘an *in vitro* and *in vivo* probe’ for CYP2A6 (16). 

Since CYP2A6 has been identified as the human coumarin 7-hydroxylase and it is responsible for the clearance of many drugs and environmental chemicals, in this study, the activity of coumarin hydroxylase as a marker of the activity of CYP2A6 is investigated in an Iranian population. 

## Materials and Methods

One hundred and fifty one volunteers (97 male, 54 female), not receiving any drug before or after the test, aged between 19 and 63 (Mean±SD= 23.8±6.6), participated in the study after giving written informed consent. Volunteers were the students or teachers of Mashhad University of Medical Sciences (MUMS), most of them students of the School of Pharmacy (east of Iran). Two of them were permanent smokers and 8 were temporary smokers. The Ethics Committee of Mashhad University of Medical Sciences approved the appropriate standards of the experiments in humans. Also, the volunteers had been given informed consent sheet prior to participating in this study.

Each volunteer took one capsule containing 5 mg coumarin (produced by department of Industrial Pharmacy of the School) with 200 ml water, after an overnight fast. A control urine sample was collected at the same time. Volunteers were allowed to drink 200 ml water 1 hr after and to eat food 3 hr after coumarin administration. Urine was voided 2, 4 and 8 hr after coumarin administration, volumes were recorded, and after centrifugation at 3000 g for 5-10 min a sample (5 ml) was frozen (- 20^o^C) until analyzed. 

Determination of 7-hydroxylation in the urine samples was performed spectrofluorometrically, essentially as described by Rautio *et al* (17). Briefly, 0.2 ml of urine samples were mixed with 0.3 ml 0.1 M acetate buffer pH 5.0 containing 100 units of β-glucuronidase from bovine liver (obtained from Sigma-Aldrich). The tubes were incubated in a metabolic shaker for 2 hr at 37^o^C. 0.05 ml of the incubate was withdrawn, mixed with 0.45 ml distilled water and 2 ml dichloromethane. The tubes were closed and shaken for 10 min. at 37^o^C. One ml of chloroform phase was taken to 2.5 ml of 0.1 M glycine buffer (pH 10.4), vortexed for 10 sec, and alkaline phase was measured immediately by a spectrofluorometer at 365 and 454 nm excitation and emission wavelengths, respectively. Standards and a sample with a known amount of 7OHC were run simultaneously. 

For the calculation of the amount and percent of total metabolized coumarin (8-hr test), the following equations was used:

$\mathrm{Metabolized coumarin}(\mathrm{mg})=\frac{7\mathrm{OHC excreted}(\mathrm{mg})\times 5}{5.54(\mathrm{mg})}$

$\mathrm{Metabolized coumarin}(\%)=\frac{\mathrm{metabolized coumarin}(\mathrm{mg})\times 100}{5}$

*Statistical analysis*

Kolmogorov–Smirnov test was used to determine the normal distribution of the data. To determine the significant difference between groups, student t-test was applied and to measure the linear correlation between variables, Pearson’s coefficient of correlation was used.

## Results

*2-hr coumarin excretion test*

**Figure 1:**
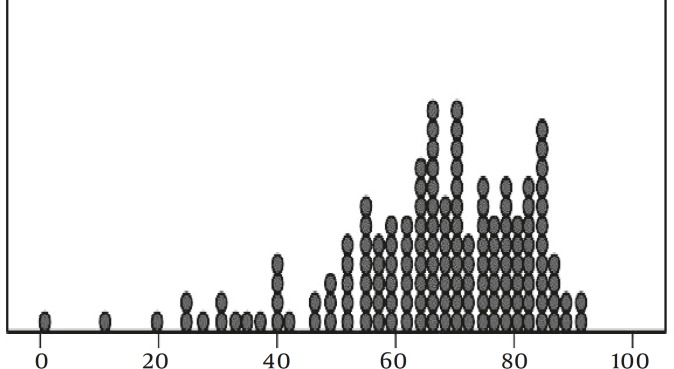
7OHC excretion in 2-hr urinary test. 150 Iranian volunteers received a 5 mg capsule of coumarin after an overnight fast and their urinary 7OHC excretion was measured spectrophotometrically.

**Figure 2:**
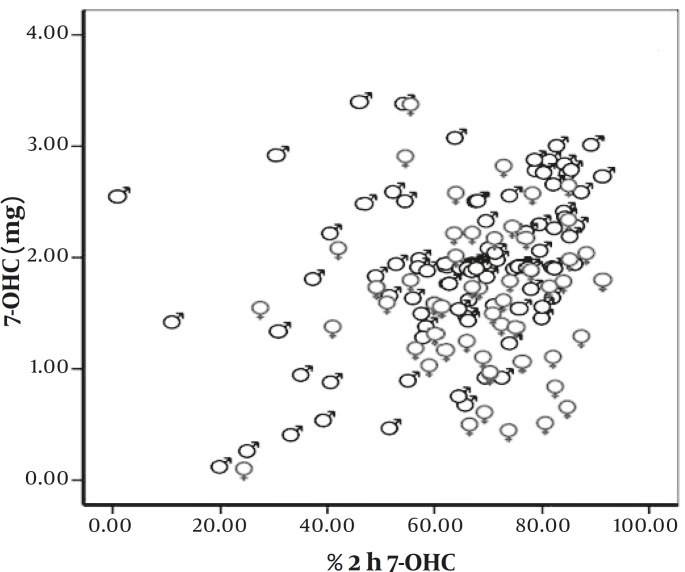
The correlation between 2-hr coumarin test value and the total amount of 7OHC excreted. Male, Female (r = 0.265, P= 0.001, n = 150).

2 hr coumarin excretion test is a basis for the comparison of the rate of CYP2A6 activity between individuals. In 150 healthy volunteers, the total amount of 7OHC recovered from urine in the first 2 hr was in the range of 0.02 and 2.68 mg (mean±SD = 1.22±0.58 mg). Table 1 shows an overall picture of the percentage of 7-OHC excretion in males and females. Figure 1 shows the results based on the percentage of total 7OHC excretion in details. There were not any significant differences between males and females in this respect. The median CYP2A6 activity was 68.62%. Kolmogorov–Smirnov test confirms the normal distribution of the data (*P=*0.064). The correlation between 2-hr coumarin test and the total amount of excreted 7OHC with sex differentiation is shown in Figure 2.

**Table 1:** Results (percent) of 2-hr 7-OH-coumarin excretion in 150 Iranian volunteers.

Volunteers	Mean	SD	Minimum	Maximum	No. of volunteers
Male	65.01%	17.80%	0.79%	91.27%	96
Female	67.73%	14.46%	24.4%	91.34%	54
Total	65.99%	16.68%	0.79%	91.34%	150

*4-hr coumarin excretion test*

Table 2 shows an overall picture of the percentage of 7-OHC excretion in males and females in the first 4 hrs. There was not any significant difference between males and females. The amount of 7OHC excreted in 4 hrs was in the range of 0.05 and 2.98 mg (mean±SD = 1.63±0.64 mg).

*8-hr coumarin excretion test*

The results are depicted in Table 3. The total amount of 7OHC recovered from urine in 8 hrs was in the range of 0.1 and 3.4 mg (mean±SD = 1.81±0.69 mg). T-test analysis implied a significant difference between males and females in the amount of 7OHC excretion. Kolmogorov–Smirnov test confirms the normal distribution of the data. Generally, 1.89% to 61.33% (mean±SD = 32.74±12.47%) of the given dose of coumarin was metabolized in these volunteers.

**Table 2:** Results (percent) of 4-hr 7-OH-coumarin excretion in 150 Iranian volunteers.

Volunteers	Mean	SD	Minimum	Maximum	No. of volunteers
Male	88.93%	8.38%	39.78%	97.53%	96
Female	89.35%	8.59%	46.32%	98.49%	54
Total	89.08%	8.43%	39.78%	98.49%	150

**Table 3:** Results (mg) of the amount of 7-OH-coumarin hydroxylase excretion in 8 hrs

Volunteers	Mean	SD	Minimum	Maximum	No. of volunteers
Male	1.92 mg	0.68 mg	0.12 mg	3.40 mg	96
Female	1.62 mg	0.67 mg	0.1 mg	3.38 mg	54
Total	1.81 mg	0.69 mg	0.1 mg	3.40 mg	150

*The effect of age and smoking*

Volunteers were divided into 2 groups, less than 30 years old and above 30. As it can be seen in Table 4, no significant statistical correlation was found between the age and the amount of 7OHC excreted in 2 hrs. Among 150 volunteers, 2 people were permanent smokers and 8 were recreational smokers. No significant difference between smokers and nonsmokers was found.

**Table 4:** Age differentiation in of 2-hr coumarin test in 150 Iranian volunteers

Volunteers age (year)	Mean	SEM	No. of volunteers
30 ≤	65.99%	17.08%	139
< 30	66.05%	10.87%	11
Total	65.99%	16.68%	150

*The effects of activity of hepatic enzymes*

In this study, the activity of aspartate aminotransferase (SGOT), alanine aminotransferase (ALT) and alkaline phosphatase of volunteers were also determined. A significant difference between the activities of these factors and the amount of the excretion of 7OHC was not found (data not shown). Determination of hepatic enzymes activity was performed to ensure that volunteers are of normal hepatic function, as decreased hepatic function will adversely affect metabolic activity of its enzymes, including CYP2A6.

## Discussion

Interindividual and interethnic differences in the activity of drug metabolizing enzymes are of great importance in drug treatment. People with significant difference in drug metabolizing enzyme activity are at higher risk of drug toxicity or poor therapeutic outcome. Cytochrome P450 enzymes are especially important in this respect, as they are responsible for the most reactions of drug metabolism. CYP2A6 (coumarin hydroxylase) is a member of this superfamily expressed in the liver. 

In this study, using coumarin 7-hydroxylation, the distribution of CYP2A6 activity in 150 volunteers, aged 19 to 63, in the province of Khorasan Razavi, Iran was investigated. As it was shown that diet has a minor effect on coumarin hydroxylase activity (18), we did not control the diet of volunteers. 

**Table 5:** Results (%) of the amount of 7-OH-coumarin hydroxylase excretion in volunteers

Volunteers	2 hr	4 hr	8 hr	No. of volunteers
Male	65.01±17.80	88.93±8.38	100	96
Female	67.73±14.46	89.35±8.59	100	54
Total	65.99±16.68	89.08±8.43	100	150

CYP2A6 is the only enzyme catalyzing this reaction and consequently the formation of 7-hydroxycoumarin can be used as ‘an *in vitro* and *in vivo* probe’ for CYP2A6 (16). The half-life of coumarin in human is 1-1.5 h (19). That is why 2-hr coumarin test is a reliable basis for the interindividual investigation of CYP2A6 activity and was used by numerous investigators *in vitro* and *in vivo*. We found a wide variation in 2-hr coumarin test in this study. The results showed a 115-fold interindividual variation of CYP2A6 activity, in the range of 0.79% to 91.27% (Table 1). There was not any significant differences between males and females, although the amount of 7OHC excretion in 8-hr test was significantly higher in males (*P*<0.01, Table 5). Another study in Iranian population found different alleles distribution compared with other populations (20). A similar study in 100 Turkish volunteers found a higher rate of CYP2A6 activity (74.7% compared with our result 65.99%) (21). A study in Finnish population (110 volunteers) also found a higher mean activity compared with our results, 84.3% compared with 65.99% (17). Chinese investigators found a 300-fold variation in coumarin hydroxylae activity with a bimodal distribution, resulting in the identification of 13.3% of volunteers as poor metabolizers (22). In this study, no breakpoint was found in coumarin hydroxylase activity, but, similar to the Chinese study, a number of volunteers with very low CYP2A6 activity were found.

Normal distribution in 2 hr coumarin excretion test implies that a phenotype differentiation could not be found. Similar conclusions were made in Turkish and Finnish studies as well. Researchers made individually defined differentiation points in coumarin hydroxylase activity. Turkish investigators defined slow hydroxylators as volunteers had a 2-hr coumarin test <40%. Finnish investigators defined this border as <60% and Chinese investigators as 47%. 8%, 28% and 10.6% of Iranian volunteers could be defined as poor metabolizers based on Turkish, Finnish and Chinese investigators, respectively. In two studies by other researchers using different markers of CYP2A6 activity in Iranian population and in different parts of the country, different rates of poor metabolizers were identified. In a study conducted on 200 Iranian volunteers in Tehran (centre of Iran), using dextromethorphan as a marker of CYP2D6 activity, poor metabolizers found to be 2.5% (23). In another study, based on genetic determination of CYP2D6 activity in Tabriz (west of Iran), 4% of the population (110 volunteers) were found to be poor metabolizers (24). 

The results presented here show the proportion of hydoxylated coumarin in 8-hr test is in the range of 1.89-61.33% (mean±SD = 32.74±12.47%). It was 60% and 65% in Turkish and Finnish populations, respectively. 

## Conclusion

Collectively, considering the results of 2-hr and 8-hr tests, a comparison between the results presented in this study with Turkish and Finnish population indicates that Iranian population shows a lower rate of coumarin hydroxylase activity. As some important carcinogens such as aflatoxin B1 and nitosamines are metabolized by CYP2A6 (8), the lower activity of CYP2A6 in Iranian population poses them to a lower risk of cancer incidence due to active metabolites of these compounds. This may also be important in the metabolism and half-life of its drug substrates such as halothane and valproic acid. As the most of our volunteers were pharmacy students, some of them from other parts of the country, we can cautiously popularize our results to Iranian population. Although for a more confident conclusion, sampling from other parts of the country is recommended in complementary studies. 
